# ePave: A Self-Powered Wireless Sensor for Smart and Autonomous Pavement

**DOI:** 10.3390/s17102207

**Published:** 2017-09-26

**Authors:** Jian Xiao, Xiang Zou, Wenyao Xu

**Affiliations:** 1 Road Traffic Intelligent Detection and Equipment Engineering Technology Research Centre, Chang’an University, Xi’an 710064, China; 2015132034@chd.edu.cn; 2 Department of Computer Science and Engineering, University at Buffalo, SUNY, Buffalo, NY 14260, USA; wenyaoxu@buffalo.edu

**Keywords:** piezoelectric effect, pavement energy harvesting, adaptive working, wireless sensor

## Abstract

“Smart Pavement” is an emerging infrastructure for various on-road applications in transportation and road engineering. However, existing road monitoring solutions demand a certain periodic maintenance effort due to battery life limits in the sensor systems. To this end, we present an end-to-end self-powered wireless sensor—ePave—to facilitate smart and autonomous pavements. The ePave system includes a self-power module, an ultra-low-power sensor system, a wireless transmission module and a built-in power management module. First, we performed an empirical study to characterize the piezoelectric module in order to optimize energy-harvesting efficiency. Second, we developed an integrated sensor system with the optimized energy harvester. An adaptive power knob is designated to adjust the power consumption according to energy budgeting. Finally, we intensively evaluated the ePave system in real-world applications to examine the system’s performance and explore the trade-off.

## 1. Introduction

Many environmental monitoring applications are used in wireless sensor networks [1,2,3,4,5] including environmental monitoring of disasters [6,7,8,9]. For such applications, one of the most difficult problems is the contradiction between the battery life of the wireless sensor network and the need for long-term monitoring. Harvesting energy from the natural environment to power wireless sensor networks has received much attention, e.g., the use of solar [10,11] or mechanical energy [12,13,14], or heat [15,16], or radio frequency (RF) [17]. While the acquisition of energy from traditional energy sources, such as solar energy, provides a potential solution the installation costs are high, maintenance is not easy and they are more affected by the environment. There is a large amount of heat stored in the road [18] but the need for roadbed temperature differences, the design and installation troubles and the effects of road activities all make energy harvesting from the road difficult. Therefore, it is ideal to use piezoelectric materials to collect vibration energy from the pavement [19,20,21,22] to supply energy to the system. Of course, piezoelectric materials have been used to provide energy for many applications, such as bridges [23,24], railways [25], reinforced concrete beam structure health monitoring [26] and so on. In this paper, piezoelectric materials are applied to the highway and used in self-powered wireless sensing.

The wireless sensor in this paper is used for road data transmission [27]. The road data monitoring system is widely applied on the highway and consumes a lot of energy. The purpose of the system is to detect road disasters, find faults ahead of routine maintenance, and to avoid traffic accidents caused by road damage. There are already some cases of road health testing, for example Mohamed Rhimi et al. [28] propose using wireless sensing technology and RF energy to drive wireless sensors buried under asphalt to transport asphalt data for the diagnosis of road health status. Guo et al. [29] consider applying simultaneous wireless information and power transfer techniques to co-operative clustered wireless sensor networks, where energy-constrained relay nodes harvest the ambient RF signal and use the harvested energy to forward the packets from sources to destinations. However, the use of RF energy requires on-board RF readers to provide energy for power supply modules and the RF energy-receiving antenna is large in size and inconvenient to bury. Therefore, the use of road piezoelectric energy has become an efficient and convenient energy source. To this end, piezoelectric pavements should harvest enough energy to supply data acquisition and wireless transmission. Obviously, road data collection and other components should be embedded into the road and will be inaccessible for frequent maintenance. In fact, this sensor system can also access different sensors to apply to other information, e.g., road traffic flow [30] or wheel tracking [31]. Therefore, a wireless sensing application for pavement energy harvesting is proposed in this paper.

This paper presents a pavement self-powered wireless sensing system that integrates a self-power module, an ultra-low-power sensor system, a wireless transmission module and a built-in power management module, ePave. The piezoelectric transducers are used to collect energy from the pressurized asphalt pavement and supply power to the sensor nodes. Because vehicle traffic is irregular, the collected energy is not uniform. Based on this feature, we run the system at intervals, depending on how much energy is collected, and the circuit that detects power is self-powered. Note that the energy collected by the vehicle over the road is very small (only a few mJ), so the internal friction of the circuit and the loss of the storage device should be very small and should be operated at low power consumption or even zero power. In summary, we make the following major contributions:

- We develop an energy harvesting device based on the vibration of the asphalt pavement at 10–20 Hz [32,33]. Our energy harvesting uses piezoelectric bimorph to convert vibrations caused by vehicle travel. The piezoelectric properties of pavements with different depths are investigated. Piezoelectric transducers are encapsulated using different materials to enhance their piezoelectric effects.
- We design a universal power management module to receive the energy generated by the piezoelectric transducer. Note that we designate an adaptive power knob that set the charge voltage to be higher than 53% of the output voltage to start supplying power to the sensing system.
- We study the internal consumption, output voltage and output energy according to different storage devices. Then we select the appropriate energy storage components to collect energy and the energy storage component is calculated according to the power consumption of the sensing system.
- We propose adaptive data acquisition and delivery modes based on the traffic volume. The working time ratio according to adaptive power knob is studied to improve the energy efficiency.
- We build a self-sustaining asphalt pavement sensing system prototype and evaluate its performance.

This paper is organized as follows. Section 2 presents some of the most relevant related work that can be found in the literature. Section 3 provides a brief introduction to the pavement sensing system and to its applications and problems. Section 4 describes some of the basic concepts of piezoelectricity and pavement piezoelectric modeling. The capacity of the energy storage element is estimated and the charging and discharging process of the system is explained. Section 5 deals with the wireless sensing, the adaptive non-timed data acquisition, the delivery scheme, and the on-time ratio for different loads under different capacitors. Section 6 presents the performance of the experimental evaluation, which is the basis for verifying the proposed pavement wireless sensor. Finally, Section 7 concludes the paper and lays out some guidelines for future work.

## 2. Related Work

It is well known that roads produce pressure, deformation, and vibration when a vehicle passes [34]. The energy dissipated in the road to mechanical energy and heat not only destroys the road surface, but also causes problems for road maintenance. Piezoelectric smart materials [34,35,36,37] have received much attention in various fields because of the ability to convert mechanical energy into electrical energy. Smart pavement applications of piezoelectric smart materials on the road to transform mechanical energy into electricity is an emerging infrastructure. With the development of science and technology, the power demand for small electronic devices (such as wireless sensors) is drastically reduced [38], making it possible to collect environmental energy for their power supply [39]. Therefore, electrical energy generated by smart pavements could apply to small electronic devices. There have been some studies on road piezoelectric and microelectronics wireless sensing but as far as we know, there are as yet no related articles on pavement piezoelectric, adaptive data acquisition, and wireless sensing.

Nizar Lajnef et al. [40] developed and validated the integrated smart sensing system and data interpretation techniques for pavement fatigue monitoring, which mainly uses the piezoelectric conversion technology and a new type of self-powered wireless sensor. The system is capable of detecting, storing and transmitting strain history and temperature for long-term monitoring. The ultimate purpose is to incorporate the traffic wander effect in the fatigue prediction algorithms. However, the self-powered wireless sensor mentioned in this paper requires the use of a falling weight deflectometer or rolling weight deflectometer to non-destructively vibrate the road surface in order to power the sensor. 

Guo et al. [41] propose a framework of joint wireless energy replenishment and anchor-point based mobile data gathering in WSNs by considering various sources of energy consumption and time-varying nature of energy replenishment, and verify the impact of utility weight and recharging rate on network performance in terms of the amount of data gathered, network utility, link flow rate and sojourn time allocation. However, the road information collection system we put forward has only the piezoelectric source of the pavement, which cannot guarantee the permanent operation of the wireless sensor, and mainly focuses on the collection of road information.

Erturk and Inman [42] presented the closed-form analytical solution for a unimorph cantilever based on the Euler–Bernoulli beam assumptions. The analytical solution is extended to bimorph configurations with series and parallel connections of piezoceramic layers that are experimentally validated. The outputs of the harvester device (e.g., current, voltage and power) are analyzed extensively for the short circuit and open circuit frequency excitations. The maximum power density is about 6.8 (mW/g^2^) cm^−3^ under a 45.6 Hz and 35 kΩ resistive load. However, the maximum power density is the peak power of unimorph cantilever. And this model only supports unimorph cantilever piezoelectric transducers and is not suitable for a piezoelectric road.

Lin and Xu [43] analyzed the effect of series matching inductance on the electromechanical properties of piezoelectric transducers. When the series matching inductor is increased, the resonance frequency is decreased, the anti-resonance unchanged, the effective electromechanical coupling coefficient is increased. The experiment proves its theoretical prediction. However, the vibration frequency on the road is very small (about 10 Hz), far less than the piezoelectric transducer resonant frequency.

Cha et al. [44] designed an underwater energy harvester that used the vibrations of a biomimetic fish tail though piezoelectric materials. The possibility of using the underwater vibration of the piezoelectric tail to power the wireless communication module was evaluated. However, the road environment is very bad and the car load is large, making it easy to crush the piezoelectric ceramics. Therefore, it is necessary to protect the piezoelectric transducer from damage and this requires sufficient power generation.

Moure et al. [45] conducted a piezoelectric test on asphalt pavement with a cymbal piezoelectric transducer and evaluated it under similar conditions as the vibrating energy collector that was wasted under normal traffic conditions. The results show that the piezoelectric cymbals in the asphalt as an energy collector is feasible and can harvest non-negligible energy. However, it has only evaluated the piezoelectric power generation by traffic flow, and there is no specific application.

Li et al. [46] designed a self-sustaining system that collects energy from the indoor environment, adapts its duty-cycle to reduce power consumption, synchronize node data transfers, and feed back to the environment through an inactive flow sensor with no energy consumption. However, due to the uncertainty of road vehicle traffic, the energy collected does not maintain a low power consumption of the system. It is necessary to transmit data non-timed based on the collected power.

The above studies have the following restrictions on rechargeable wireless sensing for smart pavement: (a) They studied the influence of the vibration frequency and the external circuit on the power generation performance of the bimorph. However, due to the influence of the natural frequency and energy storage efficiency of the pavement, the storage energy was not as large as that of the analysis; (b) Due to the impact of the road environment, the traffic load is too large for the piezoelectric transducer. Therefore, it is necessary to protect the piezoelectric transducer from the external environment and seriously reduce its life. Meanwhile, power generation is ensured; (c) Because of the uncertainty of vehicle travel, the system could not be in a state of constant power, so maintaining the low power consumption of the system is not possible. Therefore, the system can run and send data once when the storage capacity is sufficient.

## 3. The Pavement Problem and the Pavement Sensing System

We first explain the environment of the pavement problem and then give the general overview and design fundamentals of our self-powered wireless sensing system.

### 3.1. Asphalt Pavement Wireless Sensing Information Detection

Asphalt pavement produces mechanical energy and heat when a vehicle passes, and the mechanical energy can be used by the piezoelectric transducer to generate electrical energy. Most of the current road health testing uses large, cumbersome ultrasonic nondestructive testing [47] or falling weight deflectometers [48], which can be very costly in terms of both manpower and resources. Most detection instruments can only detect surface damage. It is not possible to detect the following roadbeds with invisible health hazards. The cost of road health testing often accounts for a large part of the cost of road construction, and the cost of road maintenance is rising as the road base is built.

**Figure 1:**
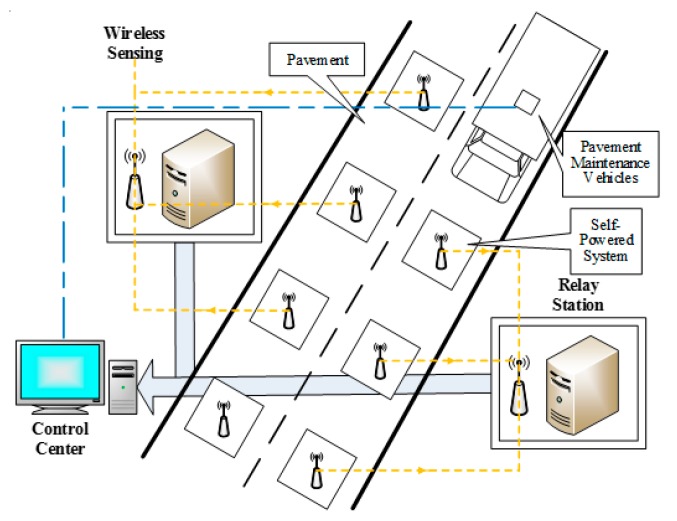
A wireless sensing information detection system for pavements.

Therefore, it is necessary to study a road health detection system that does not require man-made real-time detection and can be remotely detected and controlled, and needs to use wireless sensing to send the road information remotely to judge the road health. The system that makes up this road information is mainly composed of road environment energy harvesting and wireless sensing. Figure 1 illustrates the pavement composition and structure of this detection system.

Using a normal battery pack for wireless sensor networks [49,50] is also not possible because as they are buried in the underground system the battery cannot be replaced. It is more practical to collect the mechanical energy generated by the vehicle on the road and use it to supply power to the system. Although other environmental energy (e.g., solar, wind and heat) on the road can also be used, it is difficult to deploy on the road because of difficulties with deployment and the need for buried lines. Therefore, it is imperative to harvest pavement piezoelectric energy and provide ultra-low power wireless sensor.

In order to solve the above problems, our plan is to develop a pavement self-power wireless sensor system. We studied the power generation performance of piezoelectric ceramics based on pavement energy harvesting and on this basis we improved the energy collection efficiency and calculated the wireless sensing losses. In addition, this system is used not only to measure road information but also for other road sensor services (such as traffic flow, etc.).

### 3.2. Overview of ePave

**Figure 2:**
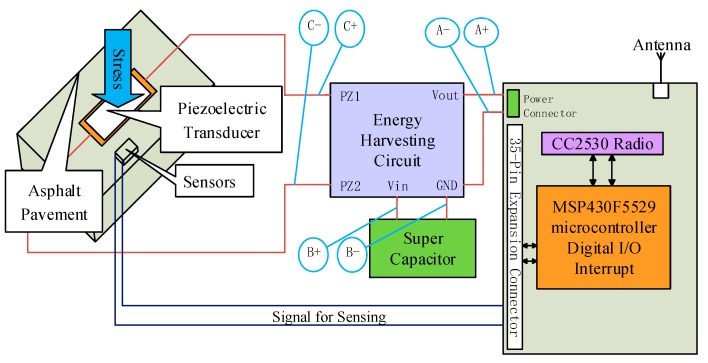
Wireless sensor system for a “smart pavement”.

Figure 2 shows the schematic of ePave. As the energy source of the system, pavement energy harvesting is placed in the left position, and the collected energy is used to run the microcontroller system as a wireless sensing platform. The receiver transmits data through the serial port to the data processing center. Two “paths” exist between the energy harvester and wireless sensing. One is used to collect the embedded asphalt concrete temperature and humidity sensor values. One is the power interface, connecting the stabilized voltage behind the energy collection circuit.

#### 3.2.1. Energy Harvesting and Power Management

**Figure 3:**
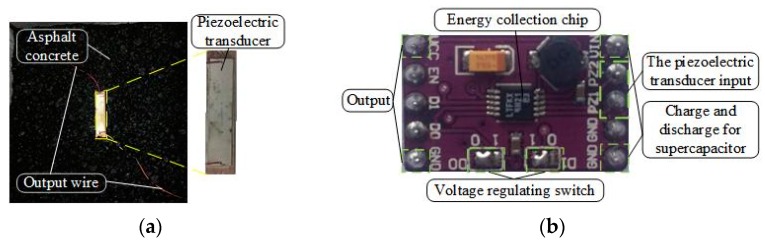
Road energy collection: (**a**) The harvester; (**b**) power management.

Our sensors are embedded in the asphalt concrete to collect data, and the piezoelectric transducers are buried inside to harvest energy. Figure 3a shows our pavement piezoelectric energy harvesting model, including asphalt concrete and piezoelectric transducers. The piezoelectric transducer used in the energy collection is buried in the center of the asphalt block.

The voltage generated by the piezoelectric transducer should not be used directly in the wireless sensing system because of its voltage spike, the voltage needs to be created smoothly through the electronic device to power the system. In general, the average power generated by the pavement (hundreds of μW) is much smaller than the power consumed by the running sensing system (mW). Therefore, it is necessary to collect the mechanical energy caused by the intermittent vehicle pressure and to study the pavement structure and how the piezoelectric coupling effect could harvest energy more efficiently. At the same time, the power consumption of the wireless sensing system should be less than or equal to the energy stored. There is the need to determine the storage of the supercapacitor so that it is enough to acquire and deliver the data required for a single chip, and the energy consumed by the wireless transmission is very large (about 120 mW). Therefore, it is necessary to calculate the capacity of the supercapacitor according to the power consumption. The power management module is shown in Figure 3b. It has three ports, one end is used to connect the output of the piezoelectric transducer, one end is used to charge and discharge the super capacitor, and the other is used to output the energy supply of the sensing system. Details on the pavement energy harvesting and power management module will be elaborated in Section 4.

#### 3.2.2. Adaptive Data Acquisition and Delivery

**Figure 4:**
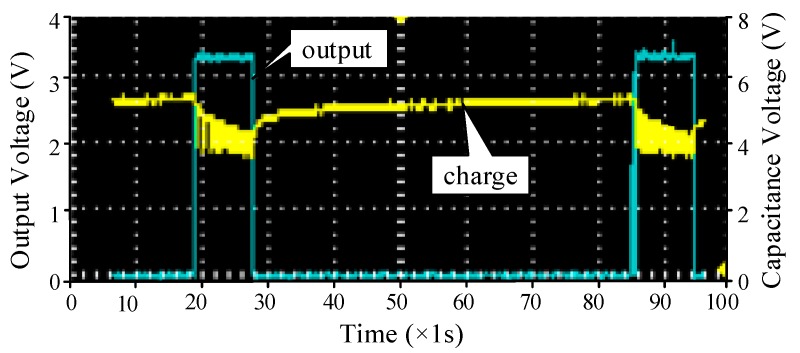
Adaptive working.

The situation of the vehicle is random and disorderly and the type of vehicle on the road that determines the mechanical energy that is input to the system is also random, so the use of piezoelectric transducers to convert energy will not be regular, so the charge time required for the super capacitor cannot be predicted. As a result, the wireless sensing system does not have enough energy to run in real time, which also causes the system to shut down and fail to periodically send data based on the timer. Therefore, we had better not need to consume power to achieve the purpose of judging the collection of energy, and to power the data acquisition and wireless transmission module periodically according to the adaptive power knob. We need to calculate the supercapacitor capacity to indirectly match the energy consumed by the wireless sensing system. In Figure 4, we assume that the load is 100 Ω, using two 1F super capacitors in the series to charge and discharge, showing the load power from the adaptive power supply, in which the blue line for the output voltage, the yellow line for the capacitor voltage across. As the capacitor voltage increases, the output voltage would stabilize to 3.3 V. After the capacitor voltage is reduced, the output is stable. After a period of discharge, the output is reduced to zero, and the supercapacitor is automatically charged. After about 58 s, the load would be powered again.

Our pavement energy harvesting depends on the weight and quantity of the vehicle. Therefore, in order to supply the system work, we use the adaptive power knob to let the system acquisition and delivery of data intermittently. Although we would like to let the system work to maintain low power consumption, and according to the power to control the acquisition of data, according to the host computer signal to send data wirelessly. But the irregular nature of the road conditions allows us to collect and transmit data intermittently. The feasibility of the adaptive program is described in detail in Section 5.

## 4. Pavement Energy Harvesting and Management

In this section, we first discuss the theory and experiments of smart pavement energy harvesting, and then tell us about the design of energy management module.

### 4.1. Piezoelectric Effect

**Figure 5:**
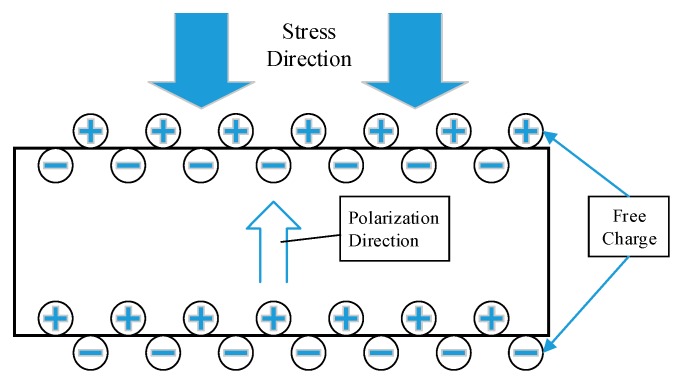
Schematic diagram of the piezoelectric effect.

The basic principle of our energy harvest is the piezoelectric effect [51,52]. Specifically, it is a smart material that converts mechanical energy into electrical energy, which changes the electric field inside the material according to stress, as shown in Figure 5.

The pavement would produce stress and vibration when the vehicle passes [33,34,35]. Piezoelectric ceramic might be a sensor or an actuator due to the positive piezoelectric effect and the inverse piezoelectric effect. The positive piezoelectric effect excites the charge on the surface of the piezoelectric material by mechanical external forces, and the charge could be stored and utilized by the energy storage device. Conventional piezoelectric materials, including piezoelectric single crystals, polycrystals, polymers and composites, have a piezoelectric effect. The piezoelectric transducers prepared in this paper are a piezoelectric polycrystal. When the piezoelectric transducer is deformed by external force, the electric dipole moment inside the material becomes shorter due to compression. At this point the polarization phenomenon causes the piezoelectric material to appear on both surfaces as the same amount of free and negative charge is released and discharge. These charges could be collected and applied. The piezoelectric effect is determined by the constitutive Equation (1) [53].

(1) $$\begin{array}{l} T_{p}=c_{pq}^{E}S_{q}-e_{kp}E_{k}\\ D_{i}=e_{iq}S_{q}+\varepsilon_{ik}^{S}E_{k} \end{array}$$

where *T*, *S*, *E*, and *D* represent the stress, strain, potential field and electrical induction of the piezoelectric transducer, respectively. $c_{pq}^{E}$ is the Young’s modulus, $e_{kp}$ is the piezoelectric coefficient, $\varepsilon_{ik}^{S}$ is the clamped permittivity. In a more general case, the open circuit voltage of the piezoelectric device can be expressed in Equation (2) of the mechanical stress in direction *p* and the induced electric field in direction *i*:

(2) $$V=T_{p}g_{ip}l.$$

Assuming that the voltage coefficient $g_{ip}$ is constant with the stress, and where *l* is the gap between the electrodes. In this paper, the use of piezoelectric transducers from PANT Technology, the specific parameters shown in Table 1.

### 4.2. Energy Harvesting

The charge generated by the piezoelectric transducer needs to be collected. The energy harvesting circuit converts the spike voltage into a stable voltage for system power supply. Specifically, we discuss the voltage under the open-circuit of the piezoelectric transducer and analyze the energy collection circuit and the energy storage unit, which is best suited for our pavement micro-energy harvesting.

#### 4.2.1. Energy Collection Interface Circuit

**Figure 6:**
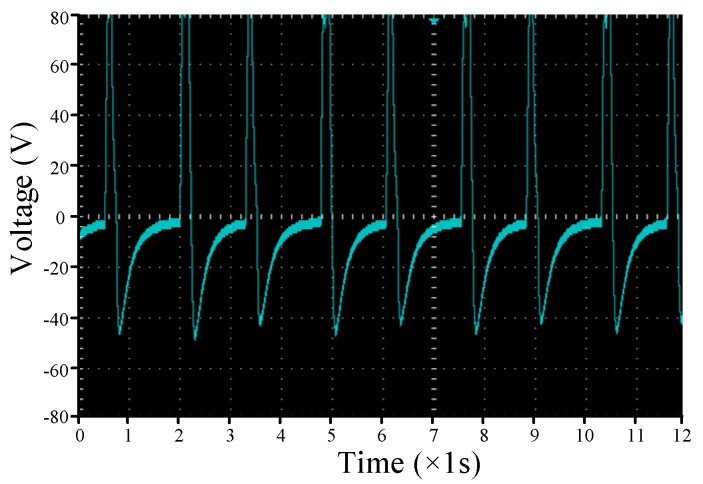
Open-circuit voltage waveform.

Figure 6 shows the open-circuit voltage waveform of the smart pavement, point C in Figure 2. It is possible to see that there is no voltage before the wheel runs over piezoelectric transducer, and when the wheel is pressed, only a peak voltage of up to 80 V is generated within 200 ms. After the wheel is left, the voltage is 40 V peak voltage on the negative half axis, and the final voltage drops to zero. This spike voltage like the alternating current is not suitable for our energy storage unit and systems, so we need a circuit that allows this voltage to become direct current.

Active and passive circuits are studied to increase the charge collection efficiency. Among the mature passive circuits is the full bridge rectifier circuit [54], but its efficiency is low. Active electric charge extraction circuits, such as synchronous electric charge extraction [55], parallel-synchronized switch harvesting inductor [56], series-synchronized switch harvesting inductor [56,57], and double synchronized switch harvesting [58], are efficient. Due to the presence of the switch and the inductance, the output voltage of the piezoelectric device is controlled, the phase difference between the output voltage and the rate is reduced, and the output power is increased. However, energy collection is difficult owing to the need to generate a switching signal and thus drive the synchronous switch. Therefore, this study uses a passive full-bridge rectifier circuit. The LT3588 is used in this study as a charge collection circuit. The LTC3588 comes with a micropower full bridge rectifier and an efficient BUCK circuit with ultra-low quiescent current undervoltage lockout (UVLO) mode allowing charge to accumulate on the input capacitor until the buck converter can effectively transfer a portion of the stored charge to the output.

#### 4.2.2. Charge Storage Unit

The charge storage unit can be a low-cost aluminum electrolytic capacitor (E-CAP) or a super capacitor (SC) with large capacity. The research on SC has been trending in recent years; its capacity is large, the volume is small, and its service life is up to ten years longer than that of the E-CAP. SC is expected to become the main power of green energy car in the future. Table 2 presents the relevant parameters of E-CAP and SC.

The energy stored in the one capacitor can be calculated by:

(3) $$E=CU^{2}/2,$$

where *E* is the energy stored in the capacitor, *C* is the capacity of the capacitor, and *U* is the voltage across the capacitor.

### 4.3. Harvesting Energy from a Smart Pavement

To estimate the power generation of a smart pavement, we need to produce, simulate and measure its storage energy under rutting conditions. We fabricated the experimental asphalt rut board. Simulation of mechanical and charge density distribution of piezoelectric transducers. And the storage energy of the piezoelectric transducer under different depth is discussed experimentally. The piezoelectric transducers are encapsulated using different protective materials, and the output energy is measured.

#### 4.3.1. Fabrication and Embedment of Piezoelectric Transducer for Asphalt Pavement

Asphalt concrete of 13 and 90 matrices is used to fabricate a rutting board. The production process is as follows.

**Figure 7:**
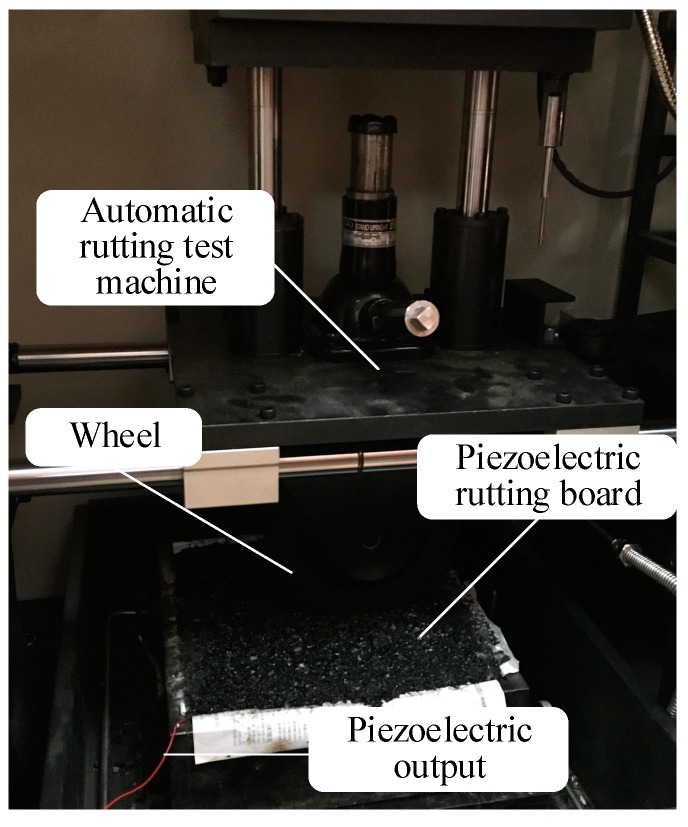
Rutting test equipment.

Aggregate, mineral powder, and asphalt are placed into a drought drying cabinet at 170 °C for two to three hours. Then, they are successively poured into the asphalt-mixing machine, in which the ratio of aggregate to asphalt is 4.6 to 5.3. The mixture is stirred at a constant temperature of 170 °C for 90 s. Next, asphalt concrete is placed into a plate specimen with 10 cm thick. Approximately half of the material is filled and flattened by hand. One piezoelectric transducer is placed on it. Fine asphalt is slowly covered in the piezoelectric transducer to avoid the stress concentration caused by large pieces of asphalt concrete. The remaining three-fifths of the material is placed into the rut plate specimen, and a piezoelectric transducer is placed on the top of it. The rest of the asphalt concrete is filled. Finally, the rutting board is placed into the rutting sample-forming machine. After adjusting the rutting board in the middle of the pressure bar, it is opened and pressed for 28 times. The pressed rutting board is cooled on the wooden frame for 12 h. The cooled rutting board is equivalent to a piezoelectric pavement. The rutting board is put into an automatic rutting test machine, with a rutting frequency of 0.7 Hz, a tire speed of 0.76 km/h, and a wheel pressure of 0.7 MPa. The finished equipment is shown in Figure 7.

The back of the piezoelectric transducer is connected to a charge-collecting circuit and a capacitor. At this point, we return to the charge-collecting circuit to access the regulator circuit, and a constant voltage is charged to the capacitor. LTC3588 chip is used to collect energy. The buried piezoelectric transducer wire is connected to the energy-harvesting circuit, and the oscilloscope probe is connected to the piezoelectric transducer and capacitor at the back to measure its electrical properties.

#### 4.3.2. Simulation Analysis of Piezoelectric Transducer

In this study, COMSOL Multiphysics software is used to simulate the piezoelectric transducers. In order to study the stress and potential distributions of piezoelectric transducers, we need to model and estimate the potential of the surface in order to determine a better experimental scheme in the application. The shape, size, and parameters used in the model are the same as those used in our experiments. Part of the parameters shown in Table 2. The piezoelectric transducers are Bimorph structures, and the thickness of the two piezoelectric ceramics is 0.5 mm. To simplify the model, cantilever structure is used, and stress of 0.7 MPa is applied. PZT and phosphor bronze are made equal. The silver electrode and binder are ignored. The position of stress and the width of the piezoelectric transducer is disregarded. We used 2D modeling for piezoelectric transducers.

**Figure 8:**
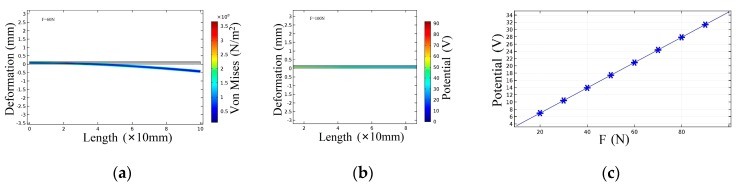
Piezoelectric transducer stress and electric potential and their relationship curve. (**a**) deformation under stress; (**b**) potential distribution; and (**c**) relationship between stress and electric potential.

The mathematical model of the COMSOL Multiphysics analysis and the effect after 60 N stress deformation are shown in Figure 8a. In order to determine the surface potential of the piezoelectric transducer under fixed stress, static analysis is used, the left end of the displacement is constrained, and the right end of the external force is activated. The cantilever can be deformed, and piezoelectric transducer will produce electric charges. The potential distribution of piezoelectric transducer at 100 N stress is shown in Figure 8b. The potential of the piezoelectric transducer decreases from left to right, and the stress of the fixed end of the piezoelectric plate is the highest. The output voltage is simulated under the condition that the length of the piezoelectric transducer is known and that the pressure is changed. The simulation result is shown in Figure 8c.

#### 4.3.3. Energy Harvesting at Different Depths

In accordance with the piezoelectric transducer being buried at different depths, the piezoelectric transducer voltage waveforms are measured through the oscilloscope probe. The piezoelectric transducer presents a peak voltage of less than 5 mV at 5 cm, and the voltage across the capacitor does not increase. Therefore, we mainly measure the voltage peak and the amount of capacitance collected at 2 cm and the surface. 

**Figure 9:**
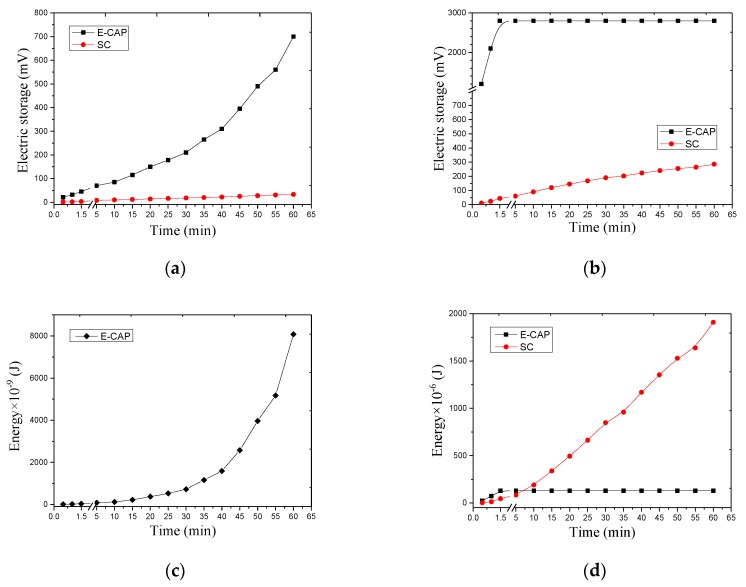
Voltage output and energy collected at different depths. (**a**) voltage at 2 cm; (**b**) voltage at the surface; (**c**) energy collected at 2 cm of E-CAP; and (**d**) Energy collected at the surface.

The voltage waveform in Figure 9a indicates that the piezoelectric transducer located at 2 cm produces a peak voltage of 1.3 V. When the oscilloscope is integrated into the capacitor, the voltage gradually and exponentially increases with time, the SC voltage is very small (less than 1 mV), and E-CAP voltage increases to 0.7 V in 1 h.

The voltage waveform in Figure 9b reveals that the piezoelectric transducer located on the surface produces a peak voltage of 23 V. When the oscilloscope is integrated into the capacitor, the SC voltage increases linearly with time, the voltage increases to 0.28 V in 1 h, and the E-CAP voltage increases to 2.8 V in 80 s.

The energy stored by the capacitor at different depths is calculated using Equation (3). We ignore the 2 cm SC value to avoid errors. Figure 9c shows that the energy collected at 2 cm in 1 h is approximately 8 µJ. The energy harvesting of E-CAP is exponentially growing.

Figure 9d presents that the energy collected by the SC in 1 h is approximately 1.9 mJ and that by E-CAP is approximately 129 µJ. E-CAP no longer stores energy from 80 s, whereas SC energy harvesting presents a linear growth over time.

The calculation indicates that the energy collected on the surface is approximately 241 times that at 2 cm. The capacitor used to collect energy on the surface should be an SC with large capacity. However, the compressive strength of piezoelectric transducer is extremely low [59] compared with asphalt concrete. Therefore, we should protect the piezoelectric transducers from damage.

#### 4.3.4. Energy Harvesting under Different Packaging Materials

Rubber, phosphor bronze, and epoxy resin are determined as packaging materials in accordance with the axle load stress of car. Part of the performance parameters of phosphor bronze and rubber material is shown in Table 3. The fabrication process for the epoxy resin packaging material is as follows: epoxy resin and polyamide resin in the ratio of 2:1 are mixed and placed at 80 °C for 10 h, and then placed at room temperature for condensation for 48 h. Epoxy resin comes from Nantong Xingchen synthetic material technology.

**Figure 10:**
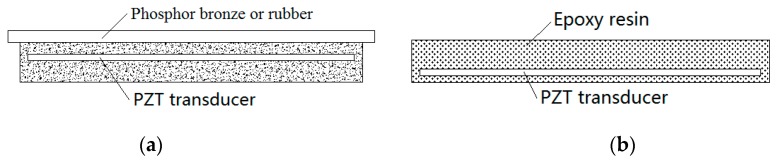
Packaging structure with different materials. (**a**) rubber and phosphor bronze; and (**b**) epoxy resin.

Given that rubber and phosphor bronze are difficult to be used to package piezoelectric transducers, asphalt sand is added. The packaging structure is shown in Figure 10a. The epoxy resin packaging structure has the same size as the rubber and phosphor bronze packages, as shown in Figure 10b.

The packaging materials are embedded on the surface of the rutted board, which is softened after heating. The rutting board is taken into an automatic rutting test machine and is connected to the circuit.

**Figure 11:**
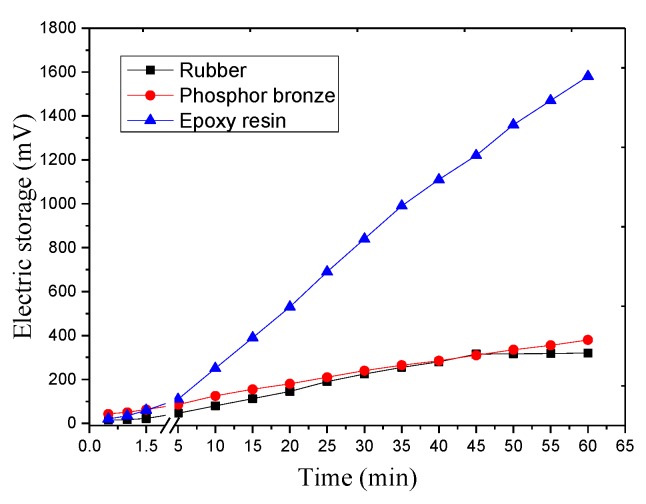
Output voltage of the different materials.

Figure 11 shows that the epoxy resin packaging material exhibits higher energy efficiency than rubber and phosphor bronze. Rubber and phosphor bronze present similar voltage output. However, after 45 min, the SC voltage of the rubber package no longer rises, and the output of the phosphor bronze as the packaging material is very stable.

**Figure 12:**
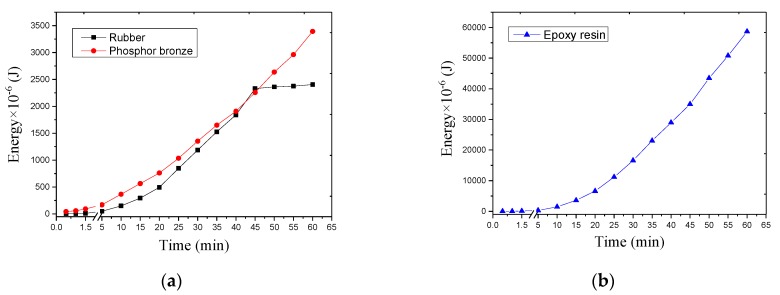
Energy collected by different materials. (**a**) rubber and phosphor bronze; and (**b**) epoxy resin.

Equation (3) is used to calculate the energy under the SC of each packaging material. Figure 12a shows the energy collected by the rubber and phosphor bronze. The energy collected by the two encapsulation materials exhibits a linear relationship with time. The rubber packaging materials no longer output energy after 45 min because the elastic modulus of rubber is very small, the elongation is extremely large, and the rubber no longer presents elastic deformation after a period of time. As a result, piezoelectric deformation is decreased. Figure 12b shows the energy collected by the epoxy resin. The energy collected by the epoxy resin as packaging material is approximately 20 times that of rubber and phosphor bronze, and the energy collection is also linearly increasing with time.

Comparison of the effects of different packaging materials on the piezoelectric transducer energy harvesting indicates that the epoxy resin is the most suitable as a piezoelectric transducer packaging material. The calculation reveals that rubber and phosphor bronze as packaging materials collect the energy of 2.4 and 3.4 mJ after 1 h, respectively. The epoxy resin as a packaging material collects energy of 58.7 mJ after 1 h. Comparison of the energy collected by a piezoelectric transducer without encapsulating material indicates that the energy collected by the piezoelectric transducer after the addition of the packaging material is significantly increased.

### 4.4. Output Power of Smart Pavement

To evaluate the power output of the intelligent pavement, we measured the output voltage of the piezoelectric transducers under open circuit and short circuit conditions and the output power under different loads. We apply the conditions in Section 4.3 to measure the optimal power output, that is, to harvest energy using a piezoelectric transducer encapsulated with epoxy resin on the pavement surface.

#### 4.4.1. Load Power under Open Circuit

**Figure 13:**
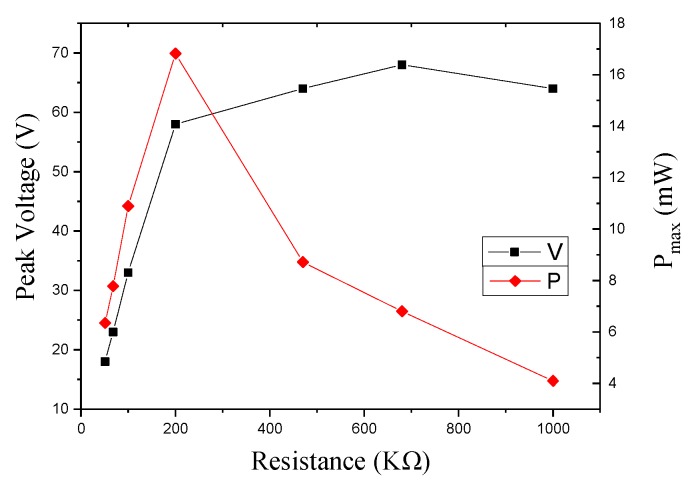
Peak voltage and output power under an open circuit.

In Section 4.2.1, we introduce the voltage characteristics of the smart pavement open circuit. We connect different load resistors to measure the output peak voltage and maximum power. Figure 13 shows the relationship between the peak voltage and the maximum power at different load resistors. With the increase of load resistance, the peak output voltage increases, and the voltage grows at 100 KΩ to 200 KΩ much greater than its growth after 200 KΩ and reaches a maximum of 68 V when the resistance is around 700 KΩ. As the load resistance increases, the output power increases first and then decreases. The output power reaches a maximum of 16.8 mW at 200 KΩ.

#### 4.4.2. Load Power under Full Bridge Rectifier Circuit

**Figure 14:**
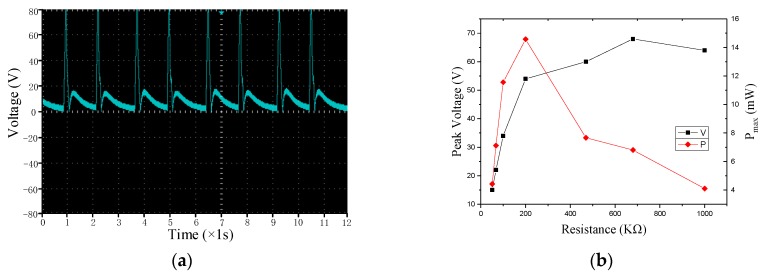
Output after full bridge rectifier circuit. (**a**) voltage waveform; and (**b**) peak voltage and power.

The function of the full bridge rectifier circuit is to convert the alternating current into direct current, such as the smart pavement voltage. The full bridge rectifier circuit is connected to different load resistances, measuring its output. As shown in Figure 14a, the voltage waveform after the full-bridge circuit is presented in two stages, and the voltage of the negative axis is reversed compared to Figure 6, and the peak voltage after inversion is reduced to 20 V. Figure 14b shows the peak voltage and maximum power after loads, and the trend is similar to that under open circuit. Peak voltage reaches a maximum of 68 V at 700 KΩ, the output power reaches a maximum of 14.6 mW at 200 KΩ.

#### 4.4.3. Load Power under Energy Harvesting Circuit

**Figure 15:**
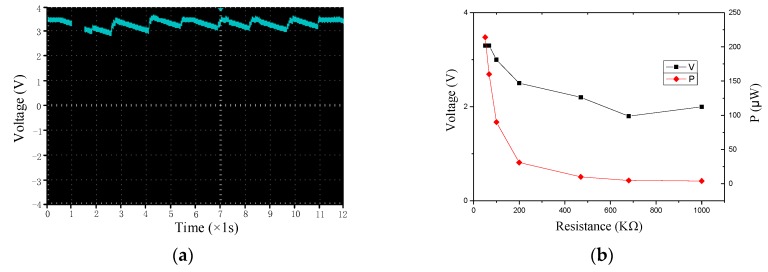
Output after energy harvesting circuit. (**a**) output voltage; and (**b**) voltage and power.

We use LTC3588 energy harvesting chip to collect the output energy of the smart pavement, in which the output voltage is measured under micro-power full bridge and voltage regulation circuit. The chip could stably output the 3.3 V voltage after a period of no load, as shown in Figure 15a. Figure 15b shows the LTC3588 output voltage after the load. As the load resistance increases, the output voltage becomes smaller and unstable, and the output power is getting lower. The output is stable at 50 KΩ load and has a maximum output power of 214 μW.

### 4.5. Regulating and Buffering Energy

From Section 4.3 and Section 4.4, we see that the peak power measured in the open circuit case reaches 16.8mW, but the energy harvested in the epoxy encapsulation is only 58.7 mJ. W = Pt of seen, 58.7 mJ of energy could be converted to 16.3 μWh, much less than the peak power. Therefore, the peak power does not represent the power output of the piezoelectric transducer. We need to store the energy and calculate the average power that could power the system.

**Figure 16:**
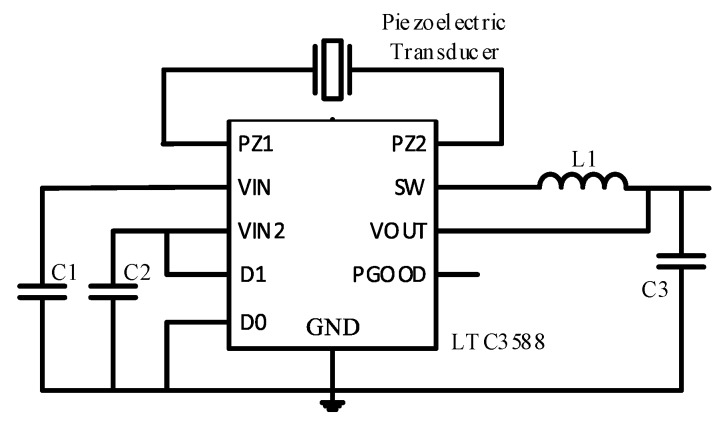
Energy harvesting circuit.

We first use the LTC3588 to collect the output power of the smart pavement and store it in the C1 super capacitor in Figure 16. The ultra-low quiescent current undervoltage lockout (UVLO) mode with the adaptive power knob allows the charge to accumulate on the input capacitor until the buck converter can effectively transfer a portion of the stored charge to the output. The chip has four output voltage can be adjusted: 1.8 V, 2.5 V, 3.3 V, 3.6 V. We adjust the microcontroller to a common voltage of 3.3 V. The UVLO range is 5.05 V–3.67 V. That is, only when the voltage on the C1 capacitor rises to 5.05 V, the buck converter is turned on and the output is stabilized by 3.3 V. When the voltage on the C1 capacitor drops to 3.67 V, the output is automatically disconnected until the super capacitor voltage is charged to 5.05 V. Chip built-in low loss full bridge rectifier, eliminating the need for an external full bridge, and further reduce the device loss.

In general, an energy management module that harvests energy from a piezoelectric transducer and supplies power to our system achieves the following three goals:

1. For the microprocessor to provide a stable 3.3 V operating voltage, which drives the acquisition module, microprocessor and wireless transmitter module work.
2. Convert the peak voltage generated by the smart pavement, and charge the direct current to the super capacitor.
3. Use the adaptive power knob to allow the stored power to release energy in stages according the energy budgeting.

## 5. Adaptive Working

As described in Section 3, the system needs to according to adaptive power knob release the power to supply the wireless sensor system. We measured the power of the ePave in the wireless sensor mode (about 120 mW) and therefore need to calculate the capacitance in the released energy so that it could have enough energy to provide the system to run. We define the concept of opening time ratio and measure the output of different energy storage capacitors and different loads. The ePave works stably according to the opening time ratio and the higher efficiency.

### 5.1. Non-Timed Work

**Figure 17:**
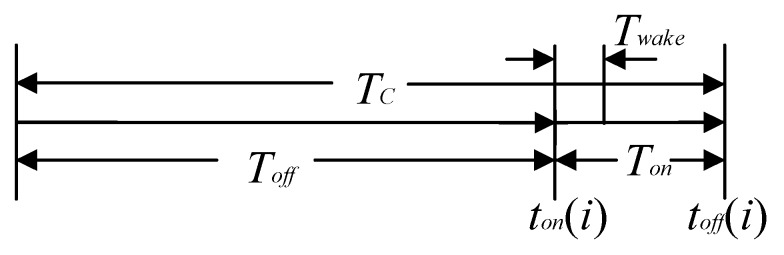
The opening and closing cycles of the sensing system.

The key to completing non-timed transmission is the charge-discharge cycle in the energy harvesting circuit. As shown in Figure 17, the non-timing operation is composed of the turn-off time Toff and the on-time Ton, and has a charging and discharging period Tc. In addition, the system usually needs to turn on Twake time to complete the initialization and power and other operations. Ron = Ton/Tc represents the on-time ratio.

Due to the serious shortage of energy supply, ePave not only needs to use the collected power to non-timed send data and need to reduce power consumption. In order to transmit data as much as possible under limited energy, we can calculate the maximum value of the Ron based on the charging power and the power consumption of the wireless sensor system. Since the disorder of the vehicle, the value of Ron is not regular, but in the case of stable input, measuring the maximum value of Ron could reduce losses and improve system efficiency.

### 5.2. Adaptive Analysis and Energy Estimation

**Figure 18:**
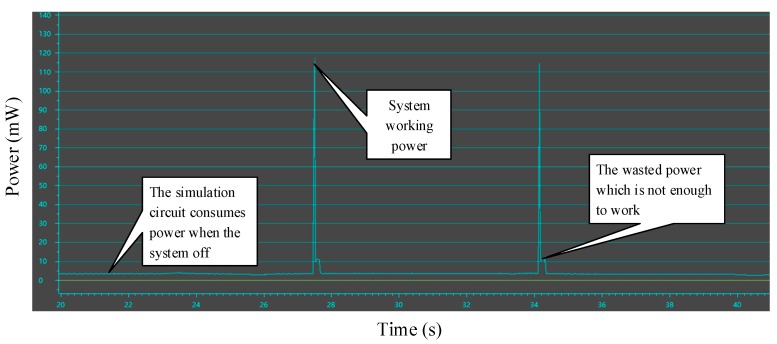
System operating power.

We calculate the power consumed by data transmission through Code Composer Studio (CCS) software. As shown in Figure 18, the working power is about 120 mW, the time is 20 ms, and the calculated energy is about 2.4 mJ. The energy stored by the capacitor could be calculated by the formula (1) to obtain a capacitance of at least 2.52 mF at a voltage drop of 5.05 V to 3.67 V. Considering the loss of efficiency and the buck converter circuit, we use the specifications of 47 mF, 0.47 F, 1F super capacitor, the two in series, as the charge storage unit to store energy according to the circuit mentioned in Section 4.5. Equivalent system load is selected for 47 Ω, 100 Ω, 150 Ω, 200 Ω, 270 Ω, 330 Ω, 470 Ω, 510 Ω, 680 Ω resistors. Connect the LTC3588 to detect the output voltage waveform to compare Ron and select the optimum efficiency conditions.

**Figure 19:**
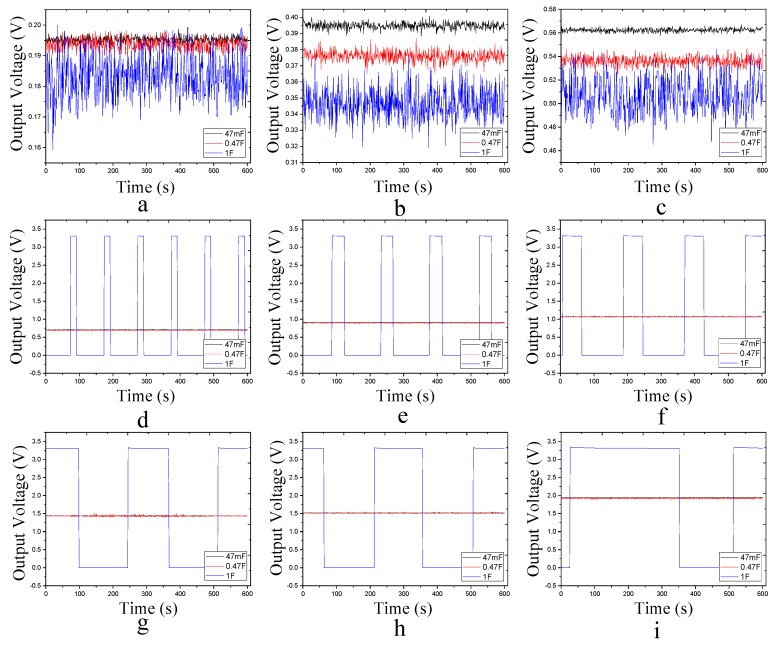
Output voltage waveforms of different loads and different capacitors. (**a**) load of 47 Ω; (**b**) load of 100 Ω; (**c**) load of 150 Ω; (**d**) load of 200 Ω; (**e**) load of 270 Ω; (**f**) load of 330 Ω; (**g**) load of 470 Ω; (**h**) load of 510 Ω; (**i**) load of 680 Ω.

Figure 19 shows the output voltage at different capacitors and different loads. In the 47 Ω, 100 Ω, 150 Ω load under the three kinds of charging the capacitor in the energy collection circuit output could not output a stable voltage. In the case where the load is larger or equal to 200 Ω, a stable output at intervals could be used only when supercapacitor with a capacity of 1F is used. We calculated the interval stability of the output of the Ron, the value shown in Table 4.

From Table 4, as the load increases, Ron also becomes large, because that the greater the resistance in the case of stable output, the smaller the output power. Therefore, reducing the power of the system could increase the value of Ron, so that improve the operating efficiency of the system. We can see that Ron has a value of 17.7 at 54.5 mW, but ePave has a running power of 120 mW, and we will evaluate the feasibility of the system below.

## 6. System Evaluations

We have established an ePave based on a smart pavement. In the following, we describe the various evaluations of this prototype. We first briefly described the deployment and setting of evaluation, and then made about the individual components, i.e. self-power module, the adaptive power management module and a wireless transmission module.

### 6.1. System Settings

**Figure 20:**
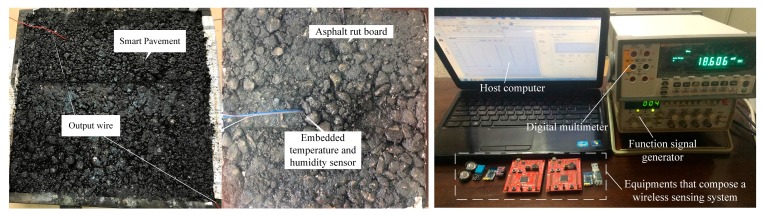
Lab setting for measurements.

Our system prototype is built using the schematic shown in Figure 20. Our hardware includes: a) smart pavement made of asphalt rutting board and piezoelectric transducer (see Figure 7b) energy management module used to process the smart pavement voltage and charge and discharge the C1 and output a stable voltage to the wireless sensing system (see Figure 3b,c) a temperature and humidity sensor (DHT11), two wireless transmission modules (CC2530) and two microprocessors (MSP430FR5529) embedded in asphalt concrete. We use the C language to implement the software part (data collection and wireless transmission) in the CCS software. In order to measure the different opening times resulting from different charging capacitors, we use FLUKE 8808A digital multimeter to monitor the voltage. Finally, we use NI LABVIEW to build the host computer and store the data sent by the wireless sensor. All of these devices are shown in Figure 20, including a smart pavement, embedded asphalt concrete temperature and humidity sensors, wireless sensing systems and monitoring equipment.

**Figure 21:**
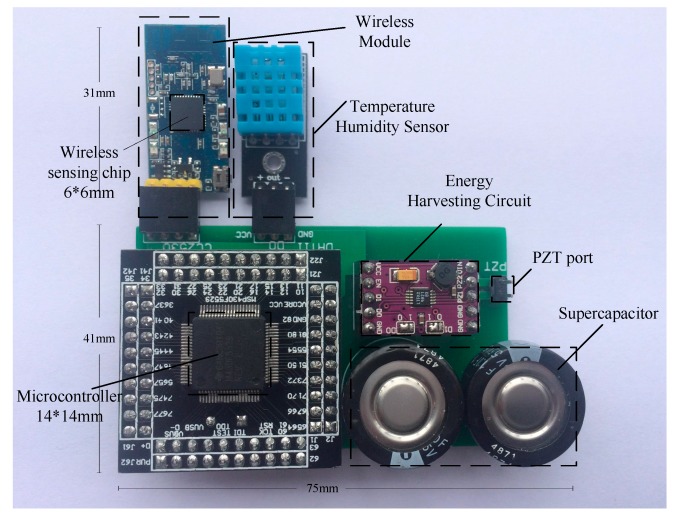
The Minimum system.

According to the hardware, we have produced the minimum system for ePave, as shown in Figure 21. The sensor is embedded in the asphalt concrete to measure the data, the use of piezoelectric transducers to the system power supply, wireless sensing module to send sensor data to the host computer, to detect the asphalt temperature and humidity or other information, in order to carry out data collection. In future tests, we will use the packaging material that does not affect the wireless data transmission and protects the system from damage to the system under the experimental asphalt concrete pavement to test the reliability of the system.

### 6.2. Energy Harvesting and Power Supplying

**Figure 22:**
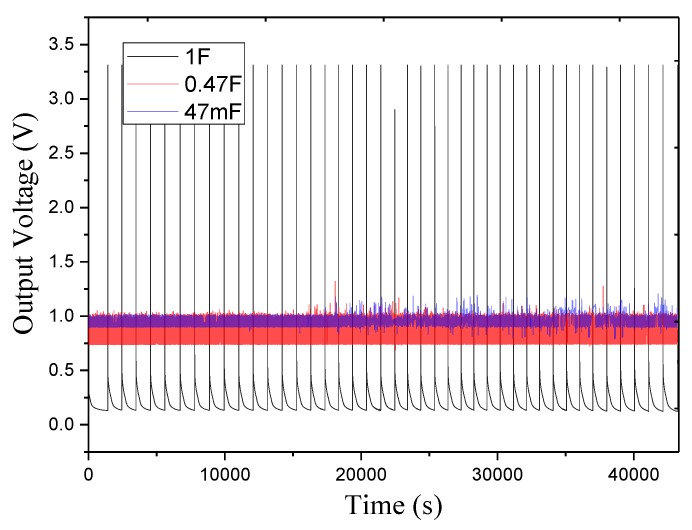
Voltage of test point A.

To observe the interaction between energy collection and system work, we use a digital oscilloscope to monitor the current and voltage at three points in the tag as shown in Figure 2. We use the function signal generator (NW1641A) to simulate smart pavement power generation. We first use Figure 22 to illustrate the voltage at point A changes. It can be seen that when using 47 mF and 0.47 F capacitor, the A point voltage is less than 3.3 V, and the ePave does not work. When the energy collection module output voltage, due to the power supply of a large internal resistance, the voltage dropped sharply to zero, then the super capacitor continues to charge, to 5.05 V after the output voltage, but a sharp drop to zero. This shows that only the theoretical calculation of 2.4 mJ energy could not bring the wireless sensing module to run. The formation of similar pulse-like energy to instantly drive the wireless sensing module, and would not have been consumed without working. When using 1 F charging capacitor, the capacitor after a period of time would be charged at the A point output 3.3 V stable voltage to provide system work. After a period of time (about 1 s) the voltage drops, the super capacitor starts charging again, waiting for the next time the system is powered.

**Figure 23:**
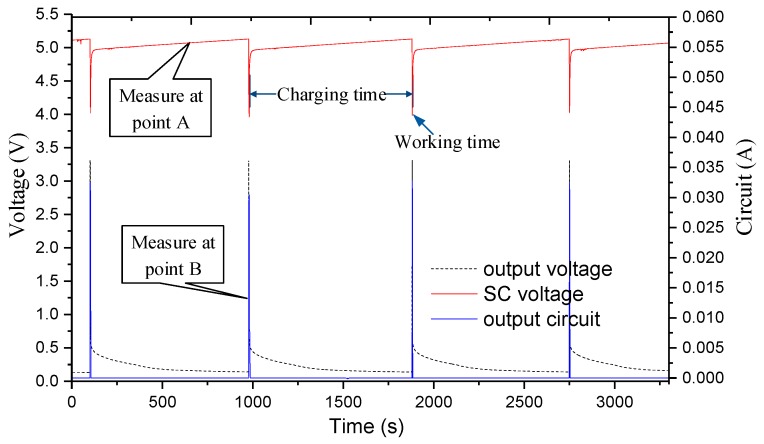
Monitoring energy harvesting and power supplying at points A and B in Figure 2.

As shown in Figure 22, the wireless sensing system operates approximately 1055 s and the opening time is about 0.05%, so we charge according to 1F capacitor and observe the data on the host computer. To evaluate the performance of the power management module accurately, we measured the voltage and current of an hour at point A and point B, as shown in Figure 23.

Figure 23 shows that the ePave running current is about 35 mA, the charging capacitor voltage drop when the energy harvesting module stable output, the peak power running about 116 mW. Our system still works after 24 h of continuous operation (in fact, ePave has been working for more than a month), but the opening time is still small, which requires us to further reduce power consumption in future work to improve the operational effectiveness.

### 6.3. Data Detection in Operation

**Figure 24:**
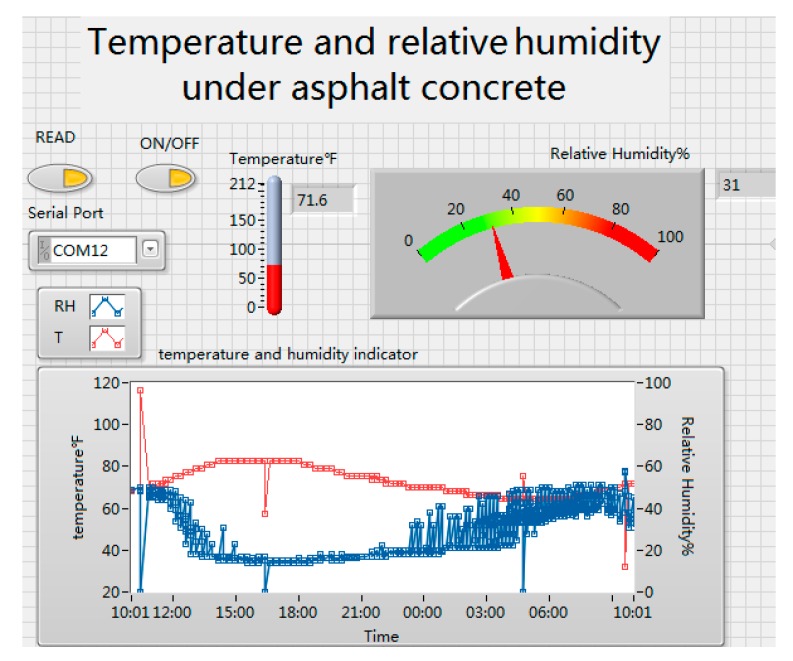
Asphalt temperature and humidity of the host computer.

Our ePave enables self-powered detection of road information. Therefore, we collected asphalt concrete temperature and humidity information through the wireless sensing system sent to the host computer, do not need to go to the field of artificial measurements, which provides a big power to the road information detecting. Figure 24 shows the change in temperature and humidity of asphalt concrete in one day, and the change in the road traffic information could be used to calculate road health information, such as rutting. We measured about 24 h of system operation. The ePave is stable operation, with good data indicators.

## 7. Conclusions and Future Work

We have proposed a self-powered wireless sensor system to facilitate smart and autonomous pavement. The ePave encapsulates three main components: self-power module, the adaptive power management module and a wireless transmission module. Harvesting energy from the environmental energy that the vehicle passes through. After the designated adaptive power knob is adjusted, the system could work stably. The ePave answers several questions that have never been completely solved. First of all, our experiments confirm that collecting low-frequency vibrations on pavements is a viable option for piezoelectric energy harvesting. Secondly, we analyze the real-time peak power on the smart pavement and test the utility of the energy harvesting module. Third, we propose an adaptive data acquisition and a delivery scheme based on the harvested energy. Finally, although the road conditions are complex and the energy harvested is limited, we have tested the wireless sensing system and displayed a meaningful pavement sensor system that relies on pavement energy harvesting and adaptive working.

In the meantime, we have tested in the laboratory of ePave, and sensor data collected 24 h embedded in asphalt concrete. However, the system is bulky and embedded difficult. We are working towards the integration of the system, which would further reduce volume and reduce power consumption. The ePAVE could be integrated into a stone shape and mixed with asphalt concrete during paving to reduce the difficulty of embedding sensors. Of course, the distance between the sensors is less than the distance from the sensor to the relay station. It could reach 150 m in such open space as the highway. Therefore, we need at least 300 m to place a relay station to transmit data. The relay station could be built into the street lights, and the received data is transmitted to the network through the cable under the street light. The control center can make decisions based on the data stored by the server. In addition, asphalt concrete sensor pavement health inspection is being studied. Different from existing pavement testing, our smart pavement wireless sensor system will be dedicated to transmitting pavement sensor information and remotely testing pavement applications.

The application of this technology in the actual pavement will be very extensive. For example, it is able to achieve remote road temperature and humidity monitoring, or realize the construction of intelligent highway by combining with the vehicle network. The monitored data can indicate or forecast potential road disasters according to the fatigue damage algorithm. Nevertheless, there are still some issues with this technology, such as the life of piezoelectric transducers, embedding orientation of sensors, transmission distance, cost, and the like. Since these issues are challenging yet solvable, in general the system could improve the pavement preservation, management and maintenance, while ultimately reducing the traffic accidents caused by road disasters.

## Figures and Tables

**Table 1:** Part parameters of piezoelectric transducers.

*d_33_*	*g_33_*	*K_p_*	*Q_m_*	*ε_r_*	*T_c_* (°C)	Size (mm)
720	20	0.62	65	4500	180	79 × 18 × 1.55

**Table 2:** Basic Information about the electrolytic capacitor (E-CAP) and super capacitor (SC).

Key Characteristics	E-CAP	SC
Nominal capacitance (F)	33 × 10^−6^	0.047
Maximum operating voltage (V)	<50	<5.5
ESR ^1^ (mΩ)	15	60
Leakage current (µA)	16.5	20
Size (mm)	Ф5 × 11	10.5 × 9.5 × 5
Endurance (h)	5000	90,000

^1^ Equivalent series resistance.

**Table 3:** Partial Parameters of Packaging Materials.

Key Characteristics	Phosphor Bronze	Rubber
Elastic modulus (Mpa)	115 × 103	7.8
Density (kg/m^3^)	8800	1800
Poisson ratio	0.35	0.47
Tensile strength (Mpa)	315	2
Elongation at break (%)	40	250
Hardness	150°	68°
Fatigue life (year)	5	>10
Size (mm)	90 × 32 × 0.5	90 × 32 × 3

**Table 4:** Output power and Ron value under different loads.

**Equivalent Load**	200 Ω	270 Ω	330 Ω	470 Ω	510 Ω	680 Ω
**R_on_**	17.7	24.8	30.9	45.4	48.9	81.1
**Output power (mW)**	54.5	40.3	33	23.2	21.4	16
